# Human Activity and Hydrogeochemical Processes Relating to Groundwater Quality Degradation in the Yuncheng Basin, Northern China

**DOI:** 10.3390/ijerph17030867

**Published:** 2020-01-30

**Authors:** Xubo Gao, Xue Li, Wanzhou Wang, Chengcheng Li

**Affiliations:** 1 State Key Laboratory of Biogeology and Environmental Geology and School of Environmental Studies, China University of Geosciences, Wuhan 430074, China; xubo.gao.cug@gmail.com (X.G.); lixue33@mail2.sysu.edu.cn (X.L.); wangwanzhou@cug.edu.cn (W.W.); 2 School of Environmental Science and Engineering, Sun Yat-sen University, No.132 Waihuan East Rd., Guangzhou University City, Panyu District, Guangzhou 510000, China

**Keywords:** human activity, groundwater quality, hydrogeochemistry, stable isotopes, Yuncheng Basin

## Abstract

Groundwater quality degradation has raised widespread concerns about water supplies and ecological crises in China. In this study, hydrogeochemistry, environmental stable isotopes (δ^18^O, δD), and principal component analysis were conducted together to reveal the mechanism’s response to the hydrogeochemical and quality degradation of groundwater in Yuncheng Basin, Northern China, so that reasonable water resource management strategies can be developed. The study reveals that groundwater faces a tremendous risk of quality decrease during the past decade: (1) the hydrochemical facies of groundwater shows that the bicarbonate and chloride type water was replaced with sulfate type water and the occupying area of SO_4_·Cl-Na, SO_4_·HCO_3_-Na type water expanded dramatically in shallow and intermediate-deep aquifers. (2) Major ion chemistry and hydrogen and oxygen isotope compositions indicate that the major hydrogeochemical processes responsible for groundwater quality deterioration include the dissolution of evaporates (i.e., halite, gypsum, and mirabilite), ion exchange, and evaporation process. Additionally, (3) anthropogenic activities (overutilization of fertilizer) have resulted in nitrate contamination, and have thereby led to groundwater quality degradation.

## 1. Introduction

Groundwater is a vital part of the total water resource for domestic, agricultural, and industrial purposes in Northern China, which is one of the world’s most water-scarce regions [1,2,3]. The study area in Yuncheng Basin, located in Shanxi province, is a typical semi-arid area in Northern China. Along with the underdeveloped surface water system, groundwater has become the primary water resource. With rapid population growth and economic development, the unreasonable exploitation and utilization of groundwater has brought about a series of environmental and geological issues such as the decrease in the groundwater level, the enlargement of the scope of groundwater salinization, the intrusion of a salt lake, and groundwater quality degradation [4,5]. Thus, a comprehensive understanding of the geochemical evolution and the factors affecting groundwater quality is crucial to guarantee the safety of groundwater consumption and the rational management of groundwater resources.

Basically, groundwater hydrogeochemistry is controlled by water–rock interactions (i.e., mineral dissolution/precipitation and ion-exchange) within the groundwater system, while human activities, such as agricultural and domestic and industrial processes, dramatically modify the groundwater chemistry [6,7]. In order to investigate the geochemical and quality evolution of groundwater, hydrochemistry, environmental isotopes (such as δ^18^O, δD), and multivariate statistical analysis have been widely used [8,9,10,11]. The hydrochemical data can effectively illustrate how the hydrogeochemical processes, such as mineral dissolution/precipitation, ion exchange, and evaporation, control groundwater quality [12,13,14]. Environmental isotopes (hydrogen and oxygen isotope) constitute effective tools to understand hydrochemical processes in the past decades, on account of their conservative characteristics [15,16,17,18].

The multivariate statistical analysis, especially principal component analysis (PCA), is an effective method to supplement the shortcomings of the traditional hydrogeochemical and isotopic tracers [19,20,21,22]. PCA can greatly simplify the influencing factors without losing much information [23,24,25].

The aim of this study is to investigate the main geochemical processes that control the hydrogeochemistry and quality degradation of groundwater under the impact of human activities using an integrated approach of hydrochemistry, environmental isotopes (δ^18^O, δD), and multivariate statistical methods. The three main objectives are to: (1) recognize and characterize the groundwater hydrochemical types; (2) investigate the main hydrogeochemical processes controlling the groundwater hydrochemical characteristics, and (3) identify the factors inducing groundwater quality degradation. The results of this study can contribute to better groundwater management and conservation in this region.

## 2. Study Area

**Figure 1:**
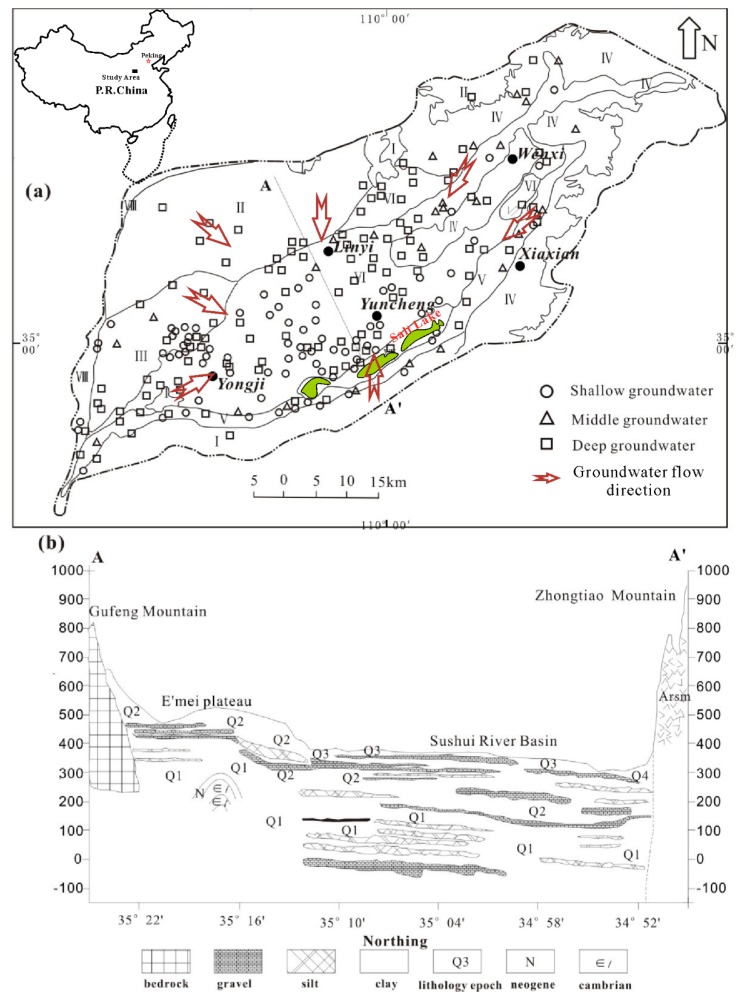
(**a**) Sampling locations at Yuncheng Basin, Shanxi Province, China. I, the Berock Mountain; II, the Emei high Uplifted area; III, the Kaolao low Uplifted area; IV, the loess hilly region; V, Piedmont plain; VI, Alluvial plain; VII, Fluvial depressions; and VIII, the Yellow River Terrace. (**b**) Schematic cross-section of the basin. Q_1_–Q_4_ represent quaternary sediments. Modified from Reference [5].

The Yuncheng Basin stretches from 34°40′ to 35°38′ N and 110°15′ E to 110°46′ E, covering an area of over 6000 km^2^ in Shanxi province (Figure 1). It was bounded in the Southeast by the frontier fault of the Zhongtiao Mountains, in the North by Emei Mountains and in the West by the Yellow River. This basin is semi-arid with an average rainfall of 550 mm/year and potential average evaporation of 1240 mm/year [26].

The basin comprises Quaternary sediments with a thickness of 300–500 m. The strata contains four major stratigraphic units (Q_1_–Q4) (Figure 1b) and is made up of inter-layered sediments, primarily aeolian loess, along with lacustrine clays, fluvial sands, and gravels. The aeolian loess mainly consists of quartz, feldspar, calcite, clays, and mica. Bedrock outcrops in the South of the basin adjacent to the Zhongtiao Mountain are Archean metamorphic rocks (Arsm). Elsewhere, the Quaternary sediments are underlying by sedimentary rocks including Neogene mudstone and Cambrian–Ordivician limestone.

The Quaternary aquifer comprises three aquifer units: a shallow unconfined unit (<70 m, Q3 and locally Q4), an intermediate semi-confined unit (70–120 m, Q2 and Q3), and a deep confined unit (>120 m, Q1 and locally Q2). Horizontally, the regional groundwater flows from the sloping margins of the basin to its flatter interior. However, on account of the excessive exploitation of groundwater for agricultural and industrial uses since the 1980s, horizontal groundwater flow is now mostly towards a cone of depression in the West of Yuncheng city. Groundwater is mainly recharged by precipitation, lateral permeation of fissure water along the basin margin, and the leakage of non-preferential river water and irrigation returns. Evapotranspiration and artificial abstraction are the major discharge ways.

## 3. Materials and Methods

A total of 183 groundwater samples, including 51 from shallow unconfined aquifers, 20 from intermediate, and 112 from deep aquifers, were collected from Yuncheng basin in September 2015 (Figure 1, Supplementary Material Table S1). The samples were stored in 250 mL polyethylene bottles that had first been pre-cleaned with deionized water in the laboratory and then with the extracted water at least three times. Before sampling, groundwater was pumped over one hour. During sampling, T, pH, electrical conductivity (EC), and oxidation-reduction potential (ORP) were measured using a portable Hanna pH and EC meter. Alkalinity was measured using the Gran titration method on the sampling day. All groundwater samples were filtered through 0.45 μm membranes on site. For cation and trace element analysis, reagent-quality nitric acid (HNO_3_) was added to the polyethylene bottles until the pH < 2. For anions analysis, the samples were stored without acidification. 

Concentrations of major cations and anions were analyzed within two weeks after sampling, using ion chromatography (IC) (ICS2100, Thermo Fisher, Massachusetts, MA, USA). Trace elements were determined by quadrupole-inductively coupled plasma-mass spectrometry (Q-ICP-MS) (Agilent 7500a ICP-MS instrument, Agilent Technologies, Tokyo, Japan). The analytical precision for all ions concentration measurements was indicated by the ionic balance error, which was better than the standard limit of 5%. δD and δ^18^O values were analyzed by an MAT 253 isotope ratio mass spectrometer (American Belemnitella from the Pee Dee formation, North California, NC, USA) at the Institute of Karst Geology, at Chinese Academy of Geological Science, based on method of gas-water equilibria, CO_2_-H_2_O for ^18^O/^16^O and H_2_-H_2_O for ^2^H/^1^H. The accuracy was ±0.1‰ and ±0.01‰ for δD and δ^18^O, respectively.

The statistical analysis (principal component analysis, PCA) of groundwater samples was performed by the statistical software SPSS 22.0 to evaluate the main factors controlling groundwater chemistry. The major ions (K^+^, Na^+^, Ca^2+^, Mg^2+^, Cl^−^, SO_4_^2−^, and HCO_3_^−^) were chosen as the analytical parameters for PCA. Factors were extracted and rotated according to the orthogonal varimax normalized rotation method [27,28].

## 4. Results

### 4.1. General Hydrogeochemistry

The major properties and hydrogeochemistry of groundwater samples were summarized in Table 1. Groundwater samples had T from 15.8 to 39 °C, with the highest temperature observed in deep groundwater samples (XX-17) from Xiaxian County, which were affected by the deep geothermal fluid [29]. pH values ranged from 6.80 and 9.02, indicating neutral to slightly alkaline conditions. Total dissolved solid (TDS) values varied from 349 and 9590 mg/L and from 205 to 14,051 mg/L for shallow and intermediate-deep groundwater samples, respectively. Over 70% of shallow groundwater and 50% of intermediate-deep groundwater belonged to the brackish or saline water category according to the classification of Reference [30]. The ionic compositions were dominated by Na^+^ (8.28–3638 mg/L), SO_4_^2−^ (11.51–9214 mg/L), and HCO_3_^−^ (15.39–1953 mg/L). According to our previous study [31], the Yuncheng Salt lake water is characterized by SO_4_·Cl-Na type with a slightly alkaline pH (7.9) and a very high TDS value (11,050 mg/L).

The hydrochemical types of both shallow and intermediate-deep groundwater displayed certain patterns along the flow path from recharge areas to flow-through and discharge areas (Supplementary Material Figures S1 and S2). Low TDS groundwater from the mountain front area was mainly HCO_3_-Na, HCO_3_-Na·Mg, and HCO_3_-Ca·Mg type. Groundwater with high TDS values collected from the center of Yuncheng Basin was generally SO_4_-Na, SO_4_·Cl-Na, SO_4_·Cl-Na·Mg, and SO_4_-Na·Mg type water. The groundwater from the flow-through areas have variable water types, including HCO_3_-Na, HCO_3_·SO_4_-Na, SO_4_·HCO_3_-Na, SO_4_·HCO_3_-Na·Mg, SO_4_·Cl-Na, and SO_4_·Cl-Na·Mg type.

The correlation analysis between major hydrochemical parameters was applied in this study (Supplementary Material Tables S2 and S3) to understand the relationships between different ionic species. EC values did exhibit a positive correlation with TDS, Na^+^, Ca^2+^, Mg^2+^, Cl^−^, SO_4_^2−^, and HCO_3_^−^, indicating that water–rock interactions played a key role in groundwater mineralization [31,32,33]. The observed well-defined correlation between TDS and Na^+^, Mg^2+^, Cl^−^, and SO_4_^2−^ suggested that processes controlling these ion compositions were, in part, related to the controls on salinity.

**Figure 2:**
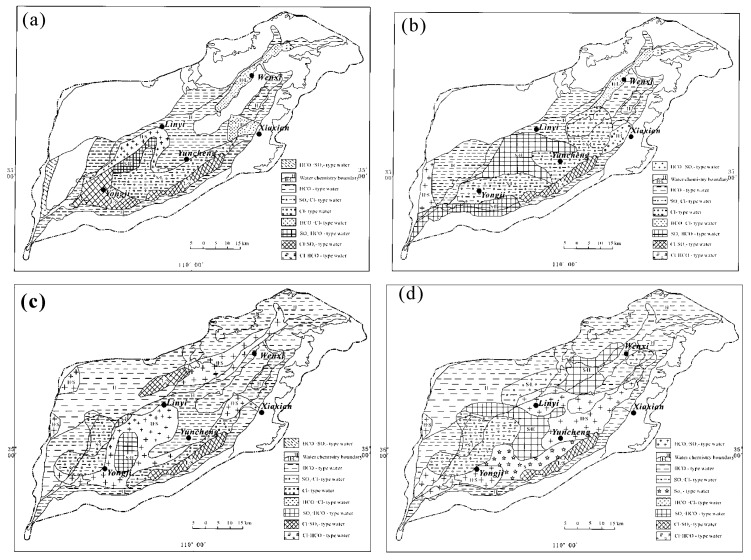
The hydrochemical facies of groundwater at Yuncheng Basin in 2005 ((**a**), shallow groundwater; (**c**), intermediate-deep groundwater) [34] and 2015 ((**b**), shallow groundwater; (**d**), intermediate-deep groundwater).

The distribution pattern of groundwater with different hydrochemical facies is illustrated for the year 2005 [34] and 2015 (Figure 2). For shallow groundwater, the major water types are HCO_3_-Na, HCO_3_-Na·Ca, HCO_3_·Cl-Na, and HCO_3_·SO_4_-Na, which are mainly found in the West and Northeast parts of the Basin in 2005 (Figure 2a). The Cl·SO_4_-Na type water are mainly presented in the surrounding areas of the Yongji city and the Salt Lake. The sulfate type water includes SO_4_·Cl-Na, SO_4_·HCO_3_-Na, SO_4_·HCO_3_-Na·Mg, and SO_4_·Cl-Na·Mg type, and scattered in the central basin between Yongji and Yuncheng city, and the belt area close to the Yellow river.

The hydrochemical facies of shallow groundwater shows a significant change in 2015, compared to that in 2005. First, the Cl·SO_4_-Na type water was replaced with the sulfate type water. Then, the occupying area of SO_4_·Cl-Na, SO_4_·HCO_3_-Na type water extended dramatically, as shown in Figure 2b. Since this sulfate type water all belongs to saline groundwater, the degradation of groundwater quality is obvious. For the intermediate-deep groundwater in Yuncheng basin, after 10 years of consumption, the distribution areas of HCO_3_-Na, HCO_3_·SO_4_, and Cl·SO_4_ type water were reduced, while the sulfate type water, SO_4_·Cl, SO_4_·HCO_3_, and SO_4_, expanded its territory (Figure 2c,d). It is regretful to see that the intermediate-deep groundwater also faces the risk of quality decrease in the area.

### 4.2. Stable Isotopes Oxygen and Hydrogen

The stable isotopic compositions of hydrogen and oxygen in the groundwater samples from Yuncheng basin are presented in Table 1. Stable isotopic composition of the shallow groundwater samples varied from −10.14‰ to −8.18‰ for δ^18^O and from −75.57‰ to −62.93‰ for δD, respectively. Intermediate and deep groundwater samples showed relatively depleted stable isotopic values, ranging from −11.34‰ to −8.36‰ for δ^18^O and from −81.87‰ to −61.80‰ for δD, respectively. 

**Figure 3:**
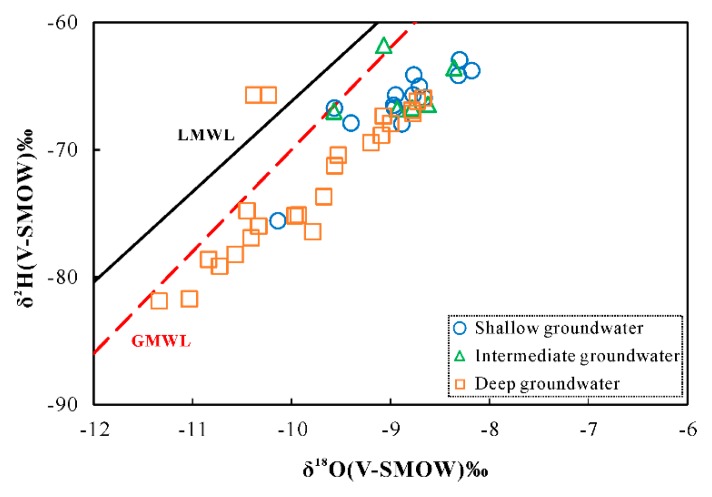
Stable isotopes δ^18^O vs. δD plot of the groundwater samples from the Yuncheng Basin, compared with the global meteoric water line (GMWL: δ^2^H = 8δ^18^O + 10 [35,36]) and local meteoric water line derived from the weighted mean monthly rainfall stable isotope values at Xi’an, 150 km Southwest of Yongji City (LMWL: δ^2^H = 7.1δ^18^O + 4.87 [37]).

The standard diagram of δ^18^O-δD diagram (Figure 3) showed the position of all groundwater samples relative to the meteoric water lines. It was observed that all the samples were plotted close to the meteoric water lines, indicating the meteoric origin in the study area. The δ^18^O and δD values for shallow groundwater defined a regression line: δD = 6.22δ^18^O − 11.51. Compared with the meteoric water lines, the relatively lower slope implied that evaporation through a dry surface layer occurred with a low moisture condition.

## 5. Discussion

### 5.1. Extraction of Principal Components by PCA

**Figure 4:**
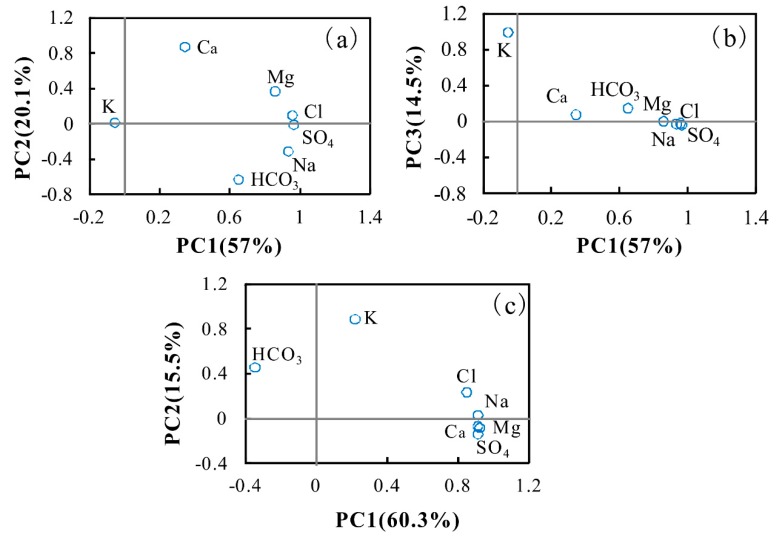
Plot of principal component loading for shallow groundwater samples (**a**,**b**) and intermediate-deep groundwater samples (**c**).

For the shallow groundwater samples, three components, accounting for 91.6% of the total variance in the dataset, were extracted (Supplementary Material Table S4). The first component (PC1) explained 57% of total variance and was represented by SO_4_^2−^, Cl^−^, Na^+^, and Mg^2+^ (Figure 4). The associations of these ions contributed to groundwater salinity and were mainly derived from evaporate dissolution. Therefore, PC1 can be seen as the impact of evaporate dissolution on groundwater chemistry. The higher the PC1 scores, the greater the impacts of evaporate dissolution. PC2 accounted for 20.1% of total variance and was characterized by high associations of Ca^2+^ and HCO_3_^−^, indicating the influence of carbonate weathering. Component three (PC3) explained 14.5% of total variance and showed a high correlation of K^+^. The source of K^+^ could be the weathering dissolution of K-bearing silicate minerals, such as K-felspar and/or cation exchange.

For the intermediate-deep groundwater samples, two principle components were extracted to explain 75.83% of the total variance (Supplementary Material Table S5, Figure 4). PC1 accounted for 60.3% of total variance with the associations of Na^+^, Ca^2+^, Mg^2+^, SO_4_^2−^, and Cl^−^, reflecting the influence of evaporate and carbonate dissolution. PC2 explained 15.55% of total variance and showed high correlations of K^+^ and HCO_3_^−^, suggesting the controlling role of K-baring silicate mineral dissolution and/or cation exchange on groundwater chemistry.

**Figure 5:**
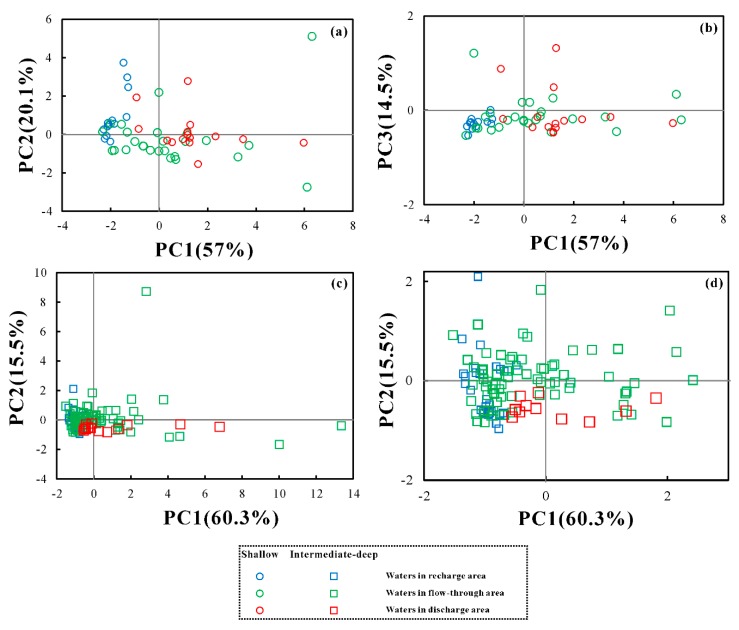
Component scores for shallow groundwater (**a**,**b**) and intermediate-deep groundwater samples (**c**,**d**).

Based on the principal component analysis, component scores for groundwater samples are calculated (Figure 5). For the shallow aquifer, the groundwater samples collected from the recharge area showed negative and uniform PC1 values (range from −2.29 to −1.27 with average −1.86), variable PC2 values (−0.37–3.74) and low PC3 values (−0.53–0.01), implying the controlling role of carbonate dissolution on groundwater chemistry in the area. The groundwater samples from the flow-through region displayed a comparatively large range for both PC1 (−2.33–6.31) and PC2 (−2.74–5.11) values, and lower PC3 values, which illustrated the influence of carbonate and evaporites dissolution on groundwater chemistry. The groundwater samples collected from the center of the basin displayed a comparatively large range PC1 values (−0.93–5.97) and smaller values for both PC2 and PC3, which highlight the potential impact of evaporate dissolution on groundwater chemistry in the center of the Yuncheng Basin.

For intermediate-deep aquifers, only two principle components (PC1 and PC2) were chosen. However, the PC1 and PC2 values of intermediate-deep groundwater samples showed a similar, changing rule. The groundwater samples collected from recharge areas showed small PC1 values (−1.37–−0.48) and higher PC2 values (−0.97–2.1), which illustrated the potential influence of silicate minerals and/or cation exchange. The PC1 and PC2 values of groundwater samples from the flow-through area are both large, which suggested the combined influence of evaporate and silicate dissolution and/or cation exchange. The groundwater samples collected from the center of the basin showed large values of PC1 and small values of PC2, which suggested that evaporate and carbonate dissolution may play dominant roles in groundwater chemistry in the area.

### 5.2. Mechanisms of Natural Processes Controlling Groundwater Chemistry

**Figure 6:**
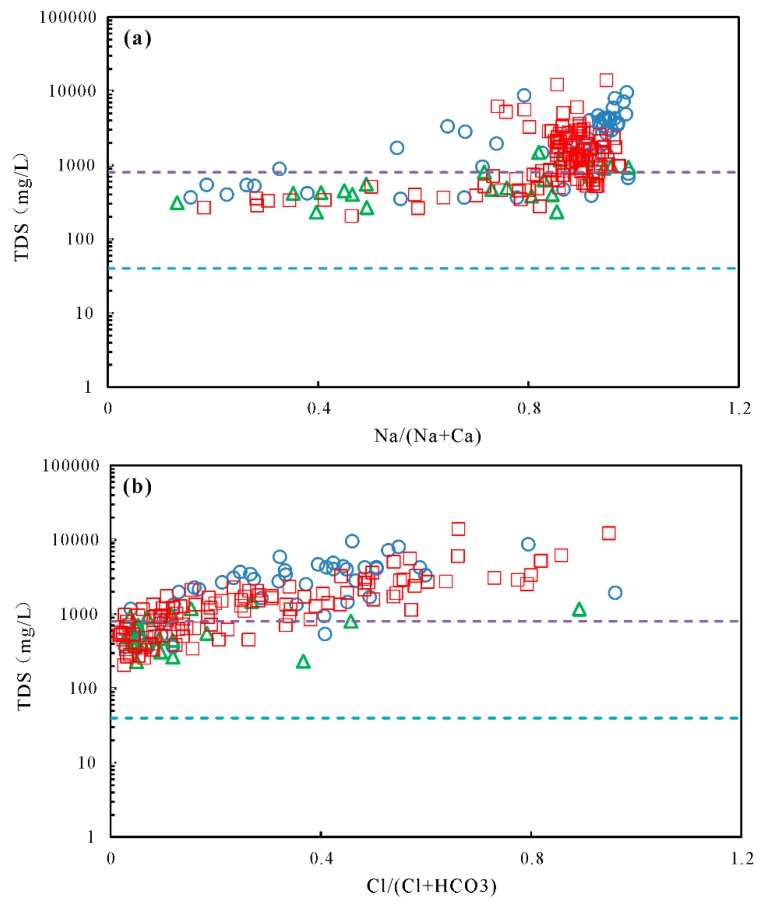
Gibbs plots of TDS vs. Na^+^/(Na^+^ + Ca^2+^) (**a**) and TDS vs. Cl^−^/(Cl^−^ + HCO_3_^−^) (**b**). Legend for groundwater samples: ○ shallow, △ intermediate, □ deep.

The Gibbs plot proved to be a useful representation to assess the natural mechanisms controlling groundwater chemistry [38,39,40,41]. In the diagram, TDS values are shown against the weight ratio of Na^+^/(Na^+^ + Ca^2+^) for cations and Cl^−^/(Cl^−^ + HCO_3_^−^) for anions (Figure 6). All the groundwater samples fell into both the evaporation and rock weathering dominant area, indicating the predominant role of evaporation and rock weathering on groundwater chemistry in the study area. Most shallow groundwater samples were positioned in the evaporation dominant zone, suggesting the significant effect of evaporation and/or dissolution of evaporates [42,43] on shallow groundwater hydrogeochemistry.

**Figure 7:**
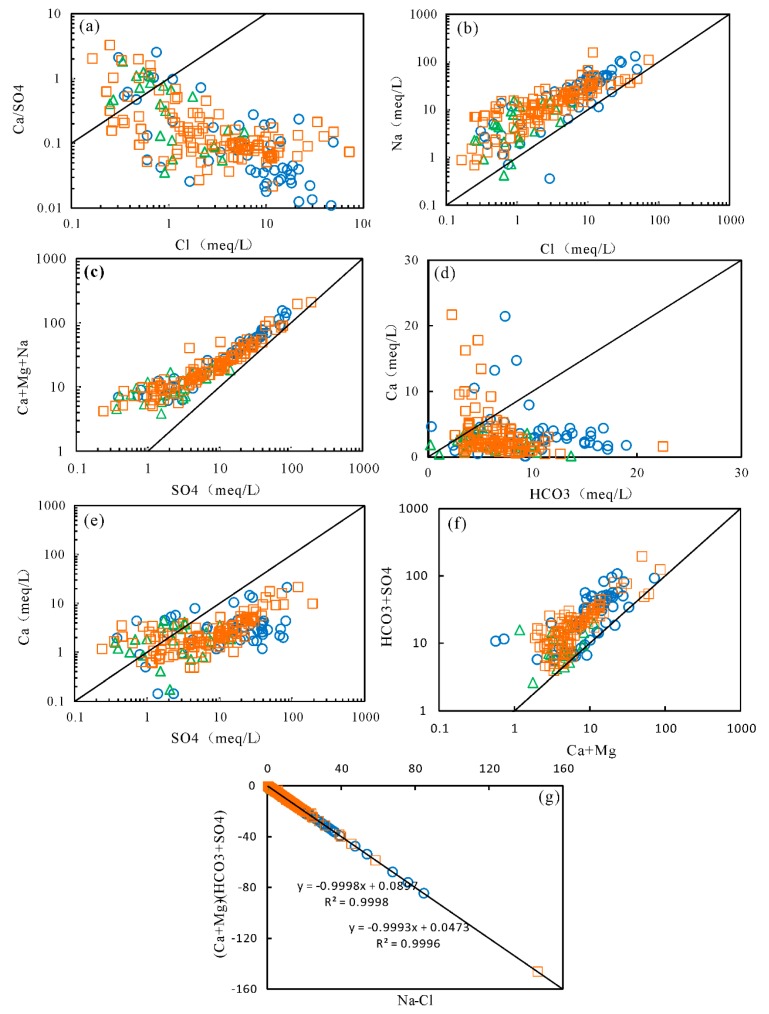
Scatter plots of major ions in groundwater; ○, shallow groundwater; ∆, intermediate groundwater; □, deep groundwater; (**a**) PCa^2+^/SO_4_^2−^ ratios versus Cl^−^, (**b**) Na^+^ versus Cl^−^, (**c**) Ca^2+^ + Mg^2+^ + Na^+^ versus SO_4_^2−^, (**d**) Plot of Ca^2+^ against HCO_3_^−^, (**e**) Plot of Ca^2+^ against SO_4_^2−^, (**f**) Ca^2+^ + Mg^2+^ versus SO_4_^2−^ + HCO_3_^−^, and (**g**) (Ca^2+^ + Mg^2+^)-(SO_4_^2−^ + HCO_3_^−^) versus Na^+^-Cl^−^. The symbols were kept the same hereafter if there are no special notes.

**Figure 8:**
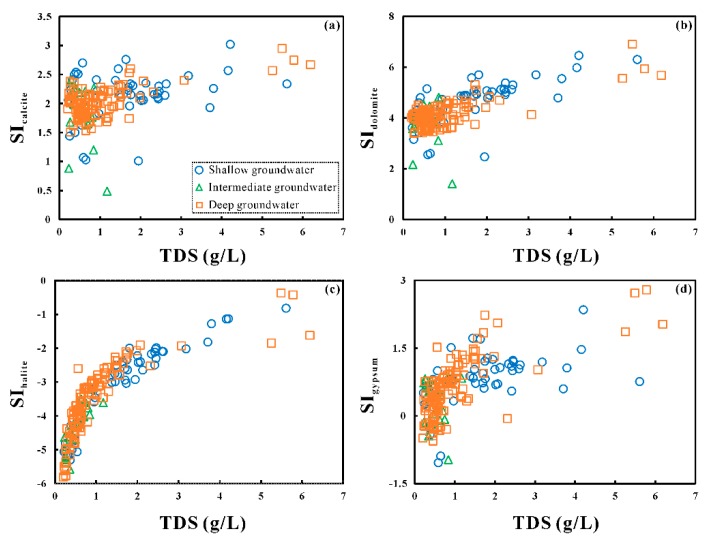
Scatter plots of TDS and four major minerals saturation index in groundwater ((**a**) calcite, (**b**) dolomite, (**c**) halite, and (**d**) gypsum).

The observed good, positive correlation between Na^+^ and SO_4_^2−^ (r = 0.87), Cl^−^ (r = 0.74) (Table S3) and the high concentrations of these major ions in shallow groundwater indicated that evaporates (i.e., halite and mirabilite [44]) dissolution was the major process controlling the shallow groundwater chemistry. The well-defined correlation between Na^+^ and SO_4_^2−^ (r = 0.93) in the intermediate-deep aquifers suggested that the dissolution of mirabilite is the major source of these ions in the groundwater. An insignificant correlation between HCO_3_^−^ and TDS contents (Table S3 and S4) was observed for both shallow and deep groundwater. Bicarbonate mainly came from carbonate and/or silicate minerals in natural waters. However, the poor correlation between HCO_3_^−^ and Ca^2+^ (Tables S3 and S4, Figure 7d) indicated that Ca^2+^ was significantly altered by other geochemical processes. The Quaternary sediments at Yuncheng Basin were made up of aeolian loess, lacustrine clays, fluvial sands and gravels, mixed with halite, mirabilite, and gypsum [4,34,45]. Combined with the low correlation between Ca^2+^ and SO_4_^2−^ in groundwater, we can conclude that gypsum was considered as one, but not the only, significant contributor to the Ca^2+^ contents. To evaluate the contribution of gypsum to calcium in groundwater, the correlation between (Ca^2+^ + Mg^2+^) and (SO_4_^2−^ + HCO_3_^−^) was illustrated in Figure 7f. If the (Ca^2+^ + Mg^2+^)/(SO_4_^2−^ + HCO_3_^−^) ratios were equal to or lower than 1, those ions were dominantly controlled by Ca-salt (i.e., calcite, dolomite, and gypsum) dissolution. The ratios higher than 1 reflected other sources for Ca, such as reverse cation exchange and/or silicate weathering [46]. As seen in Figure 7f, the majority of groundwater samples showed (Ca^2+^ + Mg^2+^)/(SO_4_^2−^ + HCO_3_^−^) ratios equal to or less than 1, suggesting the contributions of carbonate and gypsum dissolution on groundwater calcium chemistry. The positive correlations between Na^+^ and Cl^−^ (Tables S3 and S4, Figure 7b), (Ca^2+^ + Mg^2+^ + Na^+^), and SO_4_^2−^ (Figure 7c) enforced the hypothesis of the dissolution of evaporates, including halite and gypsum. Mineral saturation indices (SI) for all the groundwater samples were calculated using PHREEQC to better understand the hydrogeochemical processes that took place in the aquifer. If the groundwater was saturated with respect to a mineral, the saturation indice was expected to be positive (SI > 0) and it could have potentially precipitated this mineral out of solution. However, if the groundwater were undersaturated with respect to a mineral (SI < 0), it would continue to dissolve. The results show that all the groundwater samples were saturated with calcite and dolomite, and under-saturated with halite (Figure 8). The majority of groundwater shows a positive value of the saturation index of gypsum. The saturation indices of calcite, dolomite, halite, and gypsum show an increasing trend with increasing TDS concentrations, illustrating the major involvement of the dissolution of carbonates and evaporates.

**Figure 9:**
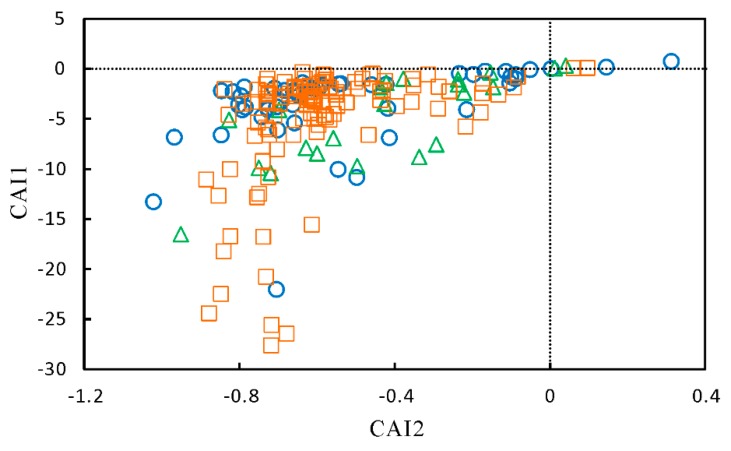
Scatter plot of CAI1 versus CAI2 for the groundwater samples in the study area.

Cation exchange was considered to be another possible factor controlling the hydrochemical compositions of groundwater at Yuncheng Basin. The plot of Na^+^ against Cl^−^ (Figure 7b) showed that some groundwater samples were positioned with a slope of 1 (halite dissolution), illustrating that Na^+^ and Cl^−^ was derived predominantly from halite. The majority of groundwater were placed above the dissolution line, which can be explained by the contribution of Na-containing minerals dissolution and/or cation exchange. The controlling role of ion exchange for the formation of groundwater chemistry can be understood by the bivariate diagram of [(Ca^2+^ + Mg^2+^)-(SO_4_^2−^ + HCO_3_^−^)] against (Na^+^-Cl^−^). The (Na^+^-Cl^−^) is related to the amount of Na^+^ gained or lost from sources other than the dissolution of chloride salts, whereas [(Ca^2+^ + Mg^2+^)-(SO_4_^2−^ + HCO_3_^−^)] showed the sum of Ca and Mg gained or lost relative to that provided by the dissolution of calcite, dolomite, and gypsum. If cation exchange was a significant factor responsible for the groundwater chemistry, the relation between [(Ca^2+^ + Mg^2+^)-(SO_4_^2−^ + HCO_3_^−^)] and (Na^+^-Cl^−^) should be linear with a slope of ± 1.0 [47]. Figure 7g depicted a straight line with a slope of −0.99 for all groundwater samples, clearly pointing out the existence of Na^+^ exchange with Ca^2+^ and Mg^2+^. Furthermore, two chloro-alkaline indices, CAI1 (CAI1 = (Cl^−^-(Na^+^ + K^+^))/Cl^−^) and CAI2 (CAI2 = (Cl^−^-(Na^+^ + K^+^))/(SO_4_^2−^ + HCO_3_^−^ + CO_3_^−^ + NO_3_^−^)) [48], were used to indicate the ion exchange between the groundwater and its host environment. The CAI1 and CAI2 values of majority of groundwater samples were negative, suggesting that ion exchange was one of the dominant processes in groundwater (Figure 9). 

**Figure 10:**
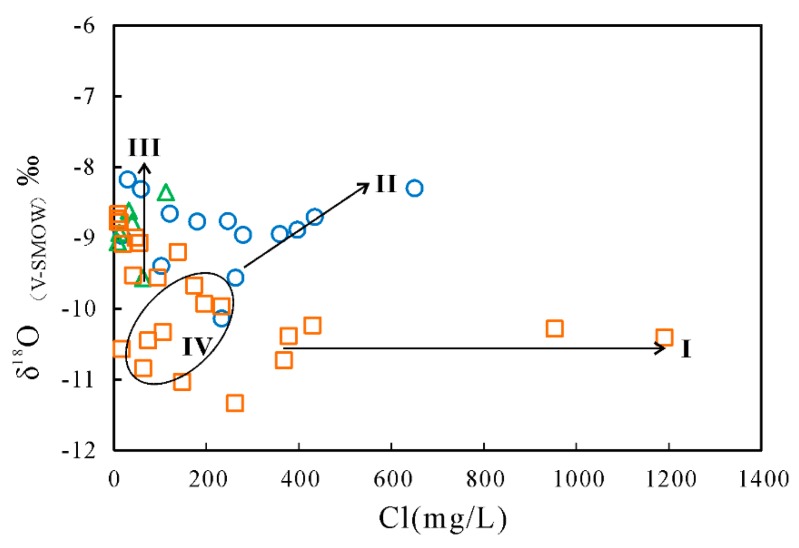
Scatter plot of δ^18^O against Cl^−^ contents for the groundwater samples in the study area.

Evaporation played an important role in the chemistry of groundwater. Judged from the Gibbs diagrams (Figure 6), the chemical compositions of some shallow groundwater were governed by evaporation process. The plot of δ^18^O and δD values further constrains the effect of evaporation on the shallow groundwater chemistry (Figure 3). The relationships between Cl and δ^18^O can also provide the potential for hydrogeochemical processes in the intermediate-deep groundwater [49,50,51,52]. From Figure 10, four trends can be detected for the groundwater samples: (I) changes in Cl contents with no accompanying variation of δ^18^O. Plenty of the deep groundwater were grouped in this group, illustrating the dissolution of evaporate during irrigation leaching [29]. During the process of leaching, little or no changes in stable isotopic composition of the deep groundwater take place due to low evaporation rates and high vertical recharge. Simultaneously, continued evaporite dissolution will lead to elevated Cl concentrations. (II) Simultaneous increase of δ^18^O and Cl values. This group included shallow groundwater samples affected by evaporation. Groundwater Cl and δ^18^O values would be expected to increase during evaporation process (i.e., recharge process or directly from the shallow water tables). (III) Changes in δ^18^O values without variation of Cl contents. The third group included plenty of deep groundwater samples and all the samples from the intermediate aquifers. The large variation in oxygen isotopic compositions with little change in Cl concentration could be due to mixing with the lateral recharge water characterized by low Cl contents and varying δ^18^O values. (IV) Slight increase in δ^18^O values with low to medium Cl contents. This group consisted of several deep groundwater, indicating the existence of an additional recharge source, probably shallow groundwater [29]. Additionally, it is interesting to note that three shallow groundwater show relatively lower Cl concentrations (< 300 mg/L) and depleted δ^18^O values (<−9.4‰). Conferring the sample numbers to their sampling site, we note that they are all collected in the flow-through and discharge areas, indicating the probable influence of up-coning of deep groundwater.

### 5.3. Anthropogenic Factors Affecting Groundwater Chemistry

Considering that Yuncheng city is one of the major crop yield areas in Shanxi Province, Northern China, agriculture activity is the dominant anthropogenic factor response for groundwater quality degradation. Due to the absence of nitrate in natural aquifers, nitrate contamination was largely due to agricultural activities. It can be observed that the NO_3_^−^ concentration of groundwater samples in the study area ranged from 1.16 to 135 mg/L. Over 15% of the shallow groundwater and 10% of intermediate-deep groundwater had NO_3_^−^ concentrations above the WHO drinking guideline (50 mg/L) [53]. Therefore, strict policies and proper management should be applied to reduce the environmental nitrate contamination.

## 6. Conclusions

Integrated analysis of hydrochemistry, environmental isotopes, and multivariate statistical analysis are applied in this study to better understand the major geochemical processes response to groundwater quality degradation in the Yuncheng Basin, Northern China.

1. Groundwater TDS values increase gradually from the mountain front recharge area to the discharge area in the central basin. Along the flow path, groundwater hydrochemical types display certain patterns of variability. The major hydrochemical facies in shallow unconfined aquifers are HCO_3_, HCO_3_·SO_4_, SO_4_·HCO_3_, SO_4_·Cl, and Cl·SO_4_-type. The major hydrochemical facies in deep confined aquifers are HCO_3_, HCO_3_·SO_4_, SO_4_·HCO_3_, SO_4_, SO_4_·Cl, and Cl·SO_4_-type. Additionally, the occupying area of SO_4_·Cl-Na, SO_4_·HCO_3_-Na type water expanded dramatically in both shallow and intermediate-deep aquifers.

2. Water–rock interactions, including the dissolution of evaporates (halite, gypsum, and mirabilite), ion exchange and evaporation, have a predominant role on groundwater quality degradation. Salt Lake water intrudes into shallow groundwater to some extent. Some deep groundwater near the faults is deteriorated by the mixing of geothermal waters.

3. Overutilization of fertilizer is another important factor response for the deterioration of groundwater quality in the study area. Reasonable strategies for fertilizer usage should be made to protect groundwater resources.

## Figures and Tables

**Table 1:** Statistical summary of hydrochemical parameters of groundwater in Yuncheng Basin.

	EC (μs/cm)	T (°C)	pH	ORP (mV)	K^+^ (mg/L)	Na^+^ (mg/L)	Ca^2+^ (mg/L)	Mg^2+^ (mg/L)	Cl^−^ (mg/L)	SO_4_^2+^ (mg/L)	HCO_3_^−^ (mg/L)	NO_3_^−^ (mg/L)	TDS (mg/L)	δ^18^O (‰)	δD (‰)
Shallow groundwater (*n* = 51)															
Min	308	15.8	6.95	−290	0.28	8.28	2.89	5.06	10.86	18.51	20.98	1.94	349	−10.14	−75.57
Max	8630	22.1	8.83	774	51.26	3024	429	605	1746	4108	1953	67.88	9590	−8.18	−62.93
Mean	2491	19	7.77	100	3.23	732	76.11	117	376	1168	631	18.17	2792	−8.9	−66.33
Intermediate groundwater (*n* = 20)															
Min	324	16.5	7.55	−30	0.52	9.75	3.53	10.89	8.86	17.51	15.39	1.16	229	−9.57	−66.96
Max	2190	22.2	8.63	734	5.59	365	89.62	92.61	213	680	836	26.56	1482	−8.36	−61.8
Mean	844	18.7	8.03	242	2.02	145	41.25	32.36	51.58	167	370	7.76	628	−8.89	−65.38
Deep groundwater (*n* = 112)															
Min	321	16.5	6.80	−95	0.26	15.73	9.78	11.67	5.85	11.51	140	1.31	205	−11.34	−81.87
Max	8860	39	9.02	873	37.69	3638	434	773	2552	9214	1373	135	14051	−8.67	−65.69
Mean	1457	20.4	7.95	157	2.36	440	64.89	64.99	221	717	401	11.91	1715	−9.84	−72.51

## Supplementary Materials

The following are available online at https://www.mdpi.com/1660-4601/17/3/867/s1; Figure S1. Piper diagram of shallow groundwater samples; Figure S2. Piper diagram of deep groundwater samples; Table S1. Groundwater sample numbers, locations and depths; results of groundwater chemical and isotopic analyses; Table S2. Pearson correlation coefficients of major hydrochemical parameter in shallow groundwater; Table S3. Pearson correlation coefficients of major hydrochemical parameter in intermediate-deep groundwater; Table S4. Variance explained by the first three principal components in shallow groundwater samples; Table S5. Variance explained by the first three principal components in intermediate-deep groundwater samples.
