# Supplementary material for: Human Activity and Hydrogeochemical Processes Relating to Groundwater Quality Degradation in the Yuncheng Basin, Northern China
Source: Int J Environ Res Public Health. 2020 Jan 30;17(3):867. doi: 10.3390/ijerph17030867 (PMC7037981; doi:10.3390/ijerph17030867)
Supplement: Supplementary file 1 [file ijerph-17-00867-s001.pdf]

International Journal of
Environmental Research and Public Health

MDPI

Supplementary Materials: Human activity and hydrogeochemical processes relating to groundwater quality degradation in the Yuncheng Basin, Northern China

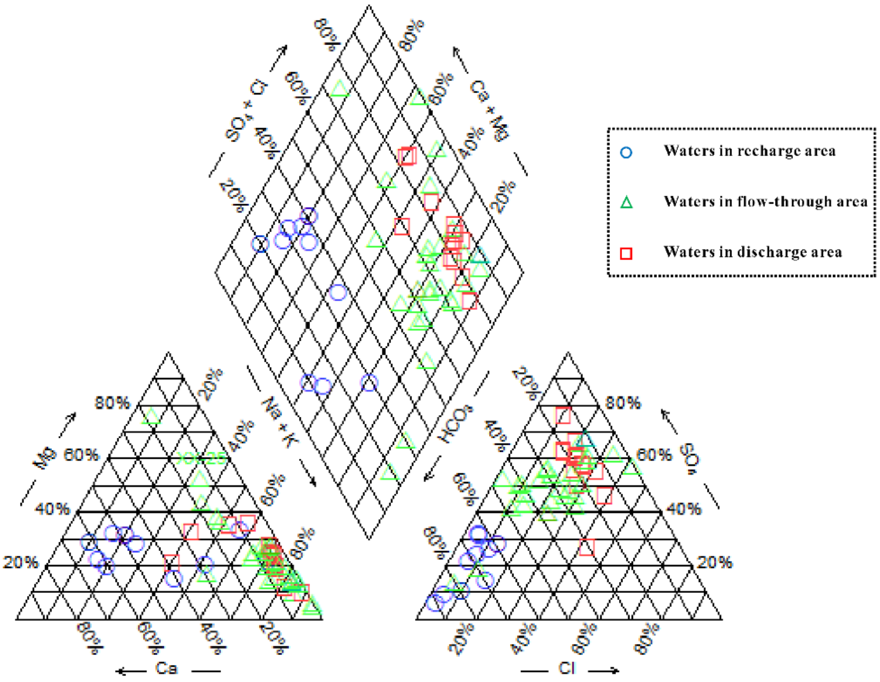

Figure S1. Piper diagram of shallow groundwater samples.

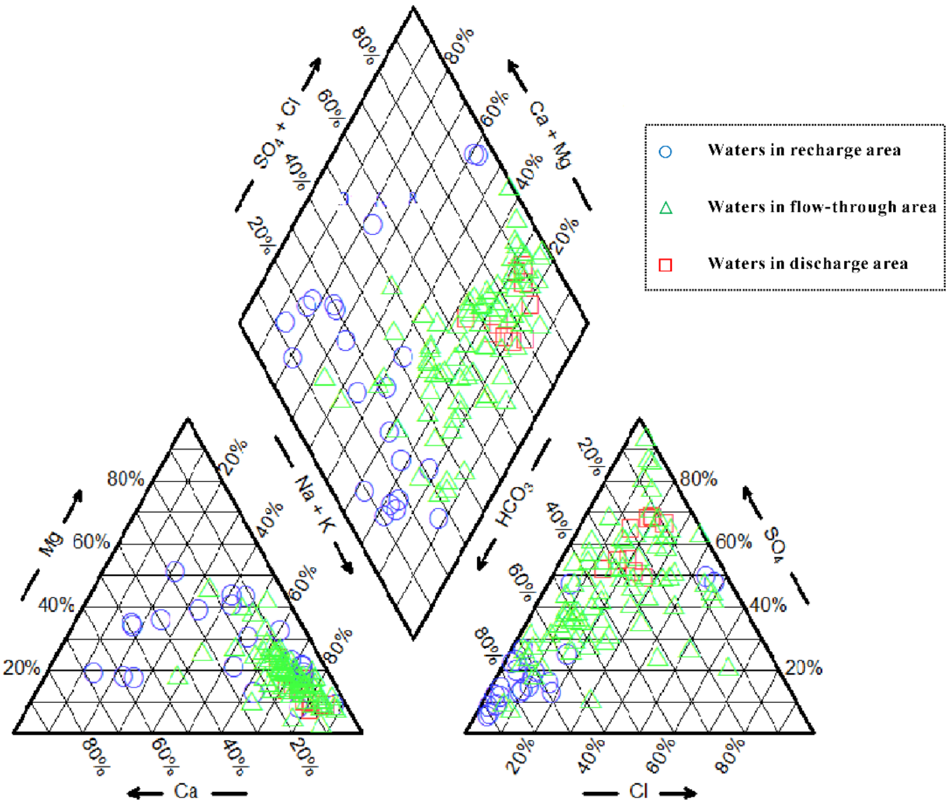

Figure S2. Piper diagram of deep groundwater samples.

International Journal of *Environmental Research and Public Health*

MDPI

Table S1. Groundwater sample numbers, locations and depths; results of groundwater chemical and isotopic analyses.

Sample No.	Unit	Longitude	Latitude	Depth （m）	T （℃）	pH	Na (mg/L)	Ca （mg/L）	Mg （mg/L）	Cl (mg/L)	SO4 (mg/L)	HCO3 （mg/L）	TDS (g/L)	stable isotope δ18O (V-SMOW)‰	δD （V-SMOW） ‰
2014YH-02	s	-	-	50	19.5	8.02	62.34	49.70	11.18	12.41	91.45	237.98	0.22	-	-
2014YH-05	s	-	-	40	22.0	7.31	256.47	210.02	69.90	263.22	757.74	270.93	0.91	-9.57	-66.72
2014YH-07	s	110º58´14"	35º00´13"	50	22.1	7.75	1134.70	52.10	199.80	633.67	1894.35	677.32	2.6	-	-
2014YH-09	s	110º57´22"	34º59´49"	20	21.5	7.85	867.26	79.76	282.44	526.43	1913.57	646.81	2.45	-	-
2014YH-10	s	110º54´26"	34º54´45"	30	19.1	7.77	26.68	91.38	19.44	21.98	84.54	305.10	0.32	-	-
2014YH-13	s	110º51´5"	34º53´44"	30	19.0	7.35	38.02	106.13	38.70	24.82	102.98	446.67	0.41	-	-
2014YH-14	s	110º46´45"	34º53´46"	20	19.8	7.14	76.99	158.88	61.64	76.22	219.79	590.67	0.57	-	-
2014YH-16	s	110º45´58"	34º53´20"	40	20.0	7.63	43.09	70.54	25.76	13.47	114.51	292.90	0.28	-	-
2014YH-21	s	110º48"44.51"	34º58"51.73"	10	19.9	7.83	1235.09	46.09	147.05	650.51	1890.51	635.83	2.44	-8.30	-62.93
2014YH-18	s	-	-	12	20.0	6.95	538.30	294.59	218.51	772.81	1246.89	517.45	1.64	-	-
2014YH-23	s	110º49´55"	34º59´15"	20	19.7	7.35	1215.93	88.18	229.69	668.23	1948.15	1025.14	3.18	-	-
2014YH-25	s	110º53´33"	34º59´41"	38	20.0	8.19	1078.09	52.10	179.62	502.50	1869.38	683.42	2	-	-
2014YH-26	s	110º54´40"	35º00´04"	28	20.0	7.63	924.97	56.51	131.01	335.00	1604.25	671.22	1.49	-	-
2014YH-30	s	110º50´44"	34º56´43"	20	19.0	7.57	1198.95	72.14	155.56	586.70	1834.80	843.30	2.63	-	-
2014YH-32	s	110º52´30"	34º57´01"	30	20.0	7.89	1593.73	23.65	90.18	771.04	1873.22	1049.54	3.71	-	-
2014YH-34	s	110º54´30"	34º57´43"	10	20.8	8.16	1139.34	80.16	82.15	363.36	1830.95	732.24	1.75	-10.14	-75.57
2014YH-36	s	110º58´10"	35º01´17"	40	19.8	7.65	1473.53	60.52	303.58	356.27	3352.58	750.55	1.81	-	-
2014YH-37	s	110º59´11"	35º02´45"	30	19.5	7.86	1140.33	57.72	204.66	476.80	2211.36	600.44	2.34	-	-
2014YH-38	s	111º4´5"	35º03´4"	20	18.0	7.86	313.38	66.13	109.38	397.93	326.61	485.72	1.7	-	-
2014YH-39	s	111º4´7"	35º03´9"	30	21.3	7.75	1178.01	71.74	160.66	767.49	1805.98	536.98	2.46	-	-
2014YH-40	s	110º59´29"	35º05´27"	12	19.0	7.61	731.92	66.13	130.04	381.09	1047.08	811.57	1.7	-	-
2014YH-41	s	110º59´36"	35º05´21"	30	19.5	7.65	762.20	41.68	108.40	246.38	1066.29	916.52	1.52	-8.76	-64.11
2014YH-50	s	111º03´41"	35º07´52"	40	18.0	7.62	553.06	56.91	72.19	120.53	776.19	811.57	0.93	-8.66	-65.90
2014LY-07	s	110º39´27"	35º02´06"	60	16.0	7.78	1074.13	34.07	88.47	358.93	1410.20	990.96	2.02	-8.95	0.66
2014LY-09	s	-	-	30	18.0	7.67	1129.77	34.07	104.52	342.98	1565.82	1050.76	2.07	-	-
2014LY-10	s	110º42´27"	35º05´32"	46	19.0	7.67	674.31	64.13	63.20	179.91	820.37	951.91	1.46	-8.77	0.66
2014LY-17	s	110º31´51"	35º00´29"	60	19.0	8.64	295.49	2.89	7.00	58.49	111.43	565.05	0.65	-8.31	-64.15
2014LY-24	s	110º47´47"	35º06´38"	30	18.0	7.60	882.29	46.09	110.84	279.17	1298.77	914.08	1.76	-8.96	-66.73
2014LY-42	s	110º30´26"	35º01´13"	60	20.0	8.83	271.74	2.89	5.06	29.42	67.63	571.15	0.59	-8.18	-63.78
2014YJ-01	s	110º35´10"	34º53´31"	50	15.8	7.15	1631.16	428.86	605.22	1745.91	4107.64	451.55	4.21	-	-
2014YJ-03	s	110º32´04"	34º52´33"	60	18.0	7.55	44.60	115.43	22.46	39.00	118.35	366.12	0.46	-	-
2014YJ-05	s	110º28´19"	34º51´51"	12	17.0	7.40	561.01	264.53	85.07	341.21	1373.70	390.53	1.45	-	-
2014YJ-13	s	110º18´23"	34º47´0"	60	18.0	8.10	16.46	88.18	24.79	26.59	34.58	347.81	0.37	-	-
2014YJ-19	s	110º15´29"	34º50´32"	50	17.0	8.11	342.70	53.23	58.63	211.99	483.39	388.09	0.83	-	-
2014YJ-20	s	110º18´12"	34º50´45"	60	19.0	7.63	601.74	76.15	54.69	192.32	708.94	951.91	1.38	-	-
2014YJ-22	s	110º21´49"	35º02´18"	60	19.0	8.17	446.39	23.25	22.85	91.11	434.97	665.12	0.97	-	-
2014YJ-23	s	110º20´39"	35º20´39"	28	17.5	7.88	1063.83	60.12	204.17	638.10	1825.19	623.62	2.42	-	-

International Journal of
Environmental Research and Public Health

MDPI

Sample No.	Unit	Longitude	Latitude	Depth （m）	T （℃）	pH	Na (mg/L)	Ca （mg/L）	Mg （mg/L）	Cl (mg/L)	SO4 (mg/L)	HCO3 （mg/L）	TDS (g/L)	stable isotope $\delta^{18}O$ (V-SMOW)‰	δD (V-SMOW) ‰
2014YJ-24	s	110º30′47"	34º59′38"	60	19.5	8.12	468.12	36.87	64.17	228.30	564.85	567.49	1.73	-	-
2014YJ-32	s	110º37′39"	34º57′02"	20	19.5	7.88	2245.83	44.09	157.99	1058.18	3246.92	945.81	3.80	-	-
2014YJ-34	s	110º37′47"	34º58′03"	20	19.0	7.53	3023.59	40.08	252.78	1659.06	3631.17	1952.64	5.61	-	-
2014YJ-37	s	110º43′09"	34º57′13"	12	20.0	7.70	2403.43	88.18	182.30	1005.01	3938.57	829.87	4.16	-	-
2014YJ-38	s	110º43′01"	34º53′54"	10	22.0	8.55	67.46	19.24	28.10	21.27	145.25	158.65	0.25	-	-
2014YJ-41	s	110º40′07"	34º52′04"	50	17.0	7.52	317.10	26.45	64.46	21.27	474.90	538.20	0.50	-	-
2014YH-B-06	s	-	-	40-50	18.0	8	633.70	88.18	118.61	397.04	941.76	671.22	2.13	-8.89	-67.97
2014LY-B-04	s	-	-	30	20.0	7.69	821.81	35.67	159.45	434.26	931.20	1159.38	2.42	-8.71	-65.00
WX-05	s	111º20′42"	35º30′5"	45	18.9	7.91	117.11	18.03	36.24	16.52	38.11	467.93	0.47	-66.5	-8.97
WX-13	s	111º18′26"	35º19′24"	25	16.8	7.99	114.81	10.02	18	39.01	45.8	291.67	0.38	-	-
XX-02	s	111º09′40.52"	35º10′30.23"	15	17.9	7.68	8.28	35.87	88.2	102.1	203.3	148.28	0.54	-67.9	-9.4
XX-04	s	111º06′50.43"	35º14′12.58"	48	17.3	7.61	149.02	60.12	85.2	187.08	318.2	273.48	0.95	-	-
XX-12	s	111º13′27.77"	35º15′29.31"	70	17.2	7.94	82.24	39.07	16.92	10.86	18.51	379.80	0.36	-	-
XX-25	s	111º08"30.40"	35º06"42.34"	50	16.8	7.47	263.16	92.98	202.3	501.8	869.5	20.98	1.95	-	-
2014YH-03	i	-	-	120	17.8	7.83	53.04	61.08	15.46	8.86	149.86	207.47	0.23	-9.07	-61.80
2014YH-04	i	-	-	110	20.4	7.71	80.56	83.37	22.46	62.75	158.31	279.47	0.41	-9.57	-66.96
2014YH-11	i	110º54′26"	34º54′45"	100	22.2	7.76	51.89	76.15	13.22	9.57	161.39	217.23	0.26	-	-
2014YH-17	i	110º46′01"	34º53′7"	110	20.0	7.82	48.85	89.62	10.89	23.04	81.46	317.30	0.29	-	-
2014YH-45	i	110º56′6"	35º10′15"	100	17.8	7.8	341.15	33.67	42.29	103.87	368.88	574.81	0.64	-	-
2014YH-54	i	111º02′24"	35º12′59"	80	18.0	7.55	327.10	72.14	92.61	212.70	484.16	579.69	0.85	-8.36	-63.56
2014YH-70	i	110º52′51"	35º10′59"	90	18.0	8.00	213.06	19.40	27.90	29.07	148.32	530.87	0.46	-	-
2014LY-02	i	110º28′5"	35º02′52"	120	19.0	8.42	365.22	3.53	11.96	32.26	99.14	835.97	0.84	-8.62	-66.44
2014LY-26	i	110º51′03"	35º08′35"	100	18.0	8.09	272.80	16.19	33.93	39.00	282.04	523.55	0.7	-8.78	-66.71
2014YJ-09	i	110º22′16"	34º50′09"	120	18.0	7.77	61.60	75.35	18.47	37.58	111.43	280.69	0.36	-	-
2014YJ-11	i	110º18′27"	34º46′01"	120	20.0	7.93	9.75	64.13	26.25	23.04	74.54	219.67	0.36	-	-
2014YJ-12	i	110º18′16"	34º44′28"	120	20.0	8.10	95.45	35.27	31.60	32.97	110.66	323.41	0.37	-	-
2014YJ-25	i	110º20′48"	34º59′36"	100	20.0	8.63	327.39	14.43	28.88	83.31	193.66	635.83	0.75	-	-
2014YJ-40	i	110º38′05"	34º52′30"	100	19.0	8.15	99.70	24.05	21.88	19.50	19.21	390.53	0.35	-	-
WX-06	i	111º20′31.6"	35º30′28"	95	18.5	7.98	180.38	37.07	27.36	31.42	47.6	608.52	0.63	-	-
WX-10	i	111º17′54"	35º26′43"	85	16.5	7.97	16.73	17.23	45.12	29.13	42.52	216.83	0.27	-	-
WX-17	i	111º10′43"	35º20′33"	90	19.0	8.44	48.01	8.21	16.2	38.49	73.56	66.45	0.23	-	-
WX-18	i	111º12′0"	35º20′26"	98	18.5	8.46	229.53	37.27	77.52	126.5	679.8	15.39	1.17	-	-
XX-05	i	111º06′40.08"	35º14′10.91"	100	16.7	8.22	127.86	50.7	67.44	145.4	315	172.76	0.80	-	-
XX-14	i	111º18′12.73"	35º13′48.65"	80	16.9	7.86	114.78	36.47	33.6	16.54	32.96	511.30	0.49	-	-
2014YH-01	d	-	-	130	19.9	7.52	58.40	41.68	36.17	28.36	63.02	336.83	0.31	-	-
2014YH-06	d	110º57′47"	34º58′52"	280	21.5	8.28	388.19	44.09	18.47	195.86	484.16	306.32	0.71	-	-
2014YH-08	d	110º58′09"	35º00′01"	320	22.5	8.15	458.66	42.08	54.69	183.45	701.26	414.94	0.79	-	-
2014YH-12	d	110º51′5"	34º53′44"	180	20.2	7.99	20.92	52.91	22.36	9.57	51.49	250.18	0.23	-	-
2014YH-15	d	110º46′45"	34º53′46"	270	19.0	7.95	86.99	37.19	18.28	41.83	53.80	296.56	0.29	-	-

International Journal of
Environmental Research and Public Health

MDPI

Sample No.	Unit	Longitude	Latitude	Depth (m)	T (℃)	pH	Na (mg/L)	Ca (mg/L)	Mg (mg/L)	Cl (mg/L)	SO4 (mg/L)	HCO3 (mg/L)	TDS (g/L)	stable isotope	
														$\delta^{18}O$ (V-SMOW)‰	δD (V-SMOW) ‰
2014YH-19	d	110º46´16"	35º00´58"	200	21.2	8.20	364.63	40.08	38.01	80.12	488.00	524.77	0.65	-	-
2014YH-20	d	110º48"44.51"	34º58"51.73"	280	20.6	8.07	242.14	32.87	40.64	94.30	292.03	416.16	0.52	-9.56	-71.26
2014YH-22	d	110º49´55"	34º59´25"	270	19.5	8.12	337.68	41.84	22.26	152.44	457.26	292.90	0.61	-	-
2014YH-24	d	110º53´33"	34º59´41"	280	19.3	7.68	297.89	48.10	57.46	98.91	500.29	421.04	0.61	-	-
2014YH-27	d	110º54´40"	35º00´04"	290	18.8	8.07	305.13	40.24	30.24	106.00	465.71	311.20	0.62	-10.33	-75.99
2014YH-28	d	-	-	280	21.7	8.01	549.69	96.99	54.20	227.77	1095.11	244.08	0.9	-	-
2014YH-29	d	110º50´44"	34º56´43"	360	20.0	8.08	712.68	101.00	78.51	367.79	1354.48	241.64	1.19	-10.73	-79.12
2014YH-31	d	110º52´30"	34º57´01"	370	20.0	7.94	555.66	55.31	34.51	199.41	968.31	244.08	0.88	-	-
2014YH-33	d	110º54´30"	34º57´43"	280	19.7	7.83	535.74	31.26	29.17	148.00	845.35	335.61	0.72	-11.03	-81.70
2014YH-35	d	110º58´10"	35º01´17"	280	18.0	7.60	975.41	112.22	97.22	398.81	1834.80	402.73	1.51	-	-
2014YH-42	d	110º59´27"	35º05´37"	240	19.8	8.01	231.45	25.65	38.89	47.15	215.18	534.54	0.55	-9.00	-67.92
2014YH-43	d	110º57´49"	35º04´50"	150	19.0	7.83	215.54	32.71	41.51	55.66	258.22	457.65	0.45	-	-
2014YH-44	d	110º55´53"	35º10´24"	160	17.0	7.7	434.65	72.62	67.28	163.07	753.13	477.18	0.76	-	-
2014YH-46	d	110º53´49"	35º07´26"	250	18.0	7.8	394.79	48.10	36.95	59.91	468.79	683.42	0.64	-	-
2014YH-47	d	110º55´36"	35º08´09"	220	17.0	7.68	403.24	60.92	55.61	186.11	583.29	475.96	0.74	-	-
2014YH-48	d	110º56´29"	35º08´52"	200	19.0	7.80	207.82	43.77	40.06	55.66	249.76	474.74	0.45	-9.07	-67.34
2014YH-49	d	111º03´13"	35º07´26"	260	19.0	7.81	283.39	32.06	30.33	57.78	221.33	622.40	0.52	-	-
2014YH-51	d	111º03´41"	35º09´41"	280	19.0	7.71	646.17	105.81	92.61	270.31	1381.38	289.23	1.04	-	-
2014YH-52	d	111º02´09"	35º11´57"	230	17.0	7.87	370.01	63.81	75.06	159.53	632.48	482.06	0.82	-	-
2014YH-53	d	111º02´31"	35º13´41"	280	18.0	7.88	1576.88	190.38	142.19	356.27	3694.57	183.06	2.07	-	-
2014YH-55	d	111º00´33"	35º13´25"	250	18.0	7.59	629.75	41.68	38.40	88.63	1068.22	488.16	0.7	-	-
2014YH-56	d	111º01´41"	35º17´42"	520	24.0	7.76	295.83	44.89	62.03	62.75	523.35	468.63	0.6	-	-
2014YH-57	d	111º00´47"	35º18´55"	380	20.0	7.73	330.40	38.48	36.46	43.25	561.01	394.19	0.41	-	-
2014YH-58	d	111º01´53"	35º19´2"	380	23.0	7.65	180.62	42.48	34.51	46.09	234.39	408.83	0.42	-	-
2014YH-59	d	110º55´55"	35º17´27"	620	23.0	7.82	455.99	59.32	59.31	222.45	733.92	380.76	0.89	-	-
2014YH-60	d	110º56´37"	35º16´0"	350	24.0	7.95	910.70	105.01	119.83	953.61	1156.59	239.20	1.98	-9.79	-76.42
2014YH-61	d	110º54´11"	35º14´36"	240	20.0	7.86	870.58	136.27	77.78	489.21	1527.40	352.70	1.46	-	-
2014YH-62	d	110º57´53"	35º16´5"	360	24.0	7.55	640.32	108.22	61.98	476.80	931.81	347.81	1.26	-	-
2014YH-63	d	110º58´23"	35º15´37"	300	22.0	8.06	477.24	30.46	33.06	164.84	495.68	616.30	0.84	-	-
2014YH-64	d	111º0´53"	35º15´56"	280	20.0	6.80	331.48	9.78	21.39	37.58	213.64	683.42	0.65	-	-
2014YH-65	d	110º59´54"	35º08´36"	160	18.0	7.78	204.49	38.64	51.63	72.67	272.82	452.77	0.52	-	-
2014YH-66	d	111º1´55"	35º10´39"	270	19.0	7.63	1236.07	324.65	175.00	290.69	3496.68	219.67	1.75	-	-
2014YH-67	d	110º59´43"	35º12´57"	250	18.0	7.95	298.93	20.04	30.53	60.27	267.44	567.49	0.59	-	-
2014YH-68	d	110º59´4"	35º12´8"	278	18.0	7.02	464.46	41.52	56.49	125.85	694.72	549.18	0.82	-	-
2014YH-69	d	110º52´30"	35º10´53"	180	23.0	7.56	756.57	140.28	79.72	755.09	1047.08	219.67	1.46	-	-
2014YH-71	d	110º54´17"	35º11´24"	258	20.0	7.76	508.26	69.26	60.08	297.78	721.62	439.34	1.03	-	-
2014YH-72	d	110º54´2"	35º10´49"	153	18.0	7.88	437.85	60.60	94.02	206.50	745.45	525.99	1.06	-	-
2014LY-01	d	110º26´56"	35º03´47"	180	24.0	8.47	348.96	9.78	18.38	21.27	188.28	774.95	0.79	-	-
2014LY-03	d	110º31´24"	35º05´50"	150	22.0	9.02	568.76	20.44	22.85	73.03	749.29	610.20	1.17	-	-

International Journal of
Environmental Research and Public Health

MDPI

Sample No.	Unit	Longitude	Latitude	Depth (m)	T (℃)	pH	Na (mg/L)	Ca (mg/L)	Mg (mg/L)	Cl (mg/L)	SO4 (mg/L)	HCO3 (mg/L)	TDS (g/L)	stable isotope $\delta^{18}O$ (V-SMOW)‰	δD (V-SMOW) ‰
2014LY-04	d	110º34´49"	35º05´15"	150	19.0	8.00	2550.76	433.67	773.42	2552.40	5859.82	140.35	5.78	-	-
2014LY-05	d	110º36´1"	35º02´19"	250	19.0	7.90	766.33	94.19	107.68	475.92	1312.22	378.32	1.66	-	-
2014LY-06	d	-	-	240	21.0	7.89	728.87	76.95	85.56	387.29	1206.55	402.73	1.51	-	-
2014LY-08	d	110º39´48"	35º00´19"	240	21.0	8.03	769.55	190.38	77.29	200.29	1898.20	256.28	1.57	-	-
2014LY-11	d	110º40´23"	35º13´02"	250	20.0	8.13	163.88	12.02	25.67	9.57	56.87	513.79	0.46	-8.73	-66.17
2014LY-12	d	110º35´15"	35º12´20"	200	19.0	7.87	163.45	17.47	28.78	9.22	110.66	475.96	0.48	-8.77	-67.13
2014LY-13	d	110º31´14"	35º13´27"	260	20.0	8.04	163.53	12.67	25.08	8.86	39.96	534.54	0.46	-8.67	-65.94
2014LY-14	d	110º33´59"	35º10´41"	285	20.0	8.35	174.58	14.43	23.53	12.41	69.93	516.23	0.49	-8.78	-66.88
2014LY-15	d	110º37´26"	35º08´32"	260	24.0	8.13	254.38	27.41	33.93	43.60	341.98	421.04	0.62	-	-
2014LY-16	d	110º32´27"	34º59´39"	260	20.0	7.53	3638.08	199.60	472.02	416.89	9214.32	213.57	5.49	-	-
2014LY-18	d	110º33´22"	35º00´15"	200	20.0	7.80	1193.30	184.37	204.17	429.83	2881.88	368.56	1.72	-10.24	0.66
2014LY-19	d	110º38´46"	35º03´7"	220	20.0	7.92	562.16	53.55	45.31	377.90	722.39	317.30	1.23	-10.38	0.66
2014LY-20	d	110º44´57"	35º07´39"	200	32.0	7.86	396.54	46.49	25.18	195.68	479.54	390.53	0.91	-9.93	-75.11
2014LY-21	d	110º43´32"	35º08´59"	200	24.0	7.70	239.26	18.44	11.67	19.50	152.93	530.87	0.55	-9.09	-68.86
2014LY-22	d	110º42´53"	35º08´11"	200	26.0	6.91	197.08	23.73	15.94	69.13	152.16	366.12	0.58	-	-
2014LY-23	d	110º45´14"	35º05´45"	230	26.0	7.84	738.64	87.37	76.08	412.11	1314.14	234.32	1.48	-	-
2014LY-25	d	110º51´50"	35º05´30"	200	18.0	7.74	366.82	55.31	55.42	137.90	499.53	555.28	0.89	-9.20	-69.43
2014LY-27	d	110º49´41"	35º09´57"	180	19.0	8.05	164.08	16.03	16.53	18.43	96.06	416.16	0.44	-	-
2014LY-28	d	110º46´24"	35º10´05"	240	30.0	7.90	473.31	49.38	25.28	292.46	585.60	292.90	1.04	-	-
2014LY-29	d	110º46´17"	35º10´28"	210	20.0	7.88	360.45	25.65	22.85	31.55	484.16	483.28	0.65	-	-
2014LY-30	d	110º46´33"	35º09´32"	130	22.0	8.00	268.52	19.24	12.64	17.73	202.88	549.18	0.59	-	-
2014YJ-02	d	110º35´07"	34º52´32"	280	19.5	8.44	179.50	30.46	14.58	79.76	179.06	278.25	0.46	-	-
2014YJ-04	d	110º30´41"	34º52´15"	178	17.8	7.70	783.50	150.70	22.36	338.90	1410.20	277.03	0.56	-	-
2014YJ-06	d	110º25´18"	34º51´20"	200	19.0	7.88	78.16	77.92	18.86	43.60	151.39	273.37	0.35	-	-
2014YJ-07	d	110º23´54"	34º51´08"	200	16.5	8.19	36.76	69.74	13.13	23.40	57.64	263.61	0.27	-	-
2014YJ-08	d	110º23´41"	34º49´44"	200	17.5	7.66	29.35	67.01	12.06	13.47	127.57	158.65	0.26	-	-
2014YJ-10	d	110º23´12"	34º52´23"	240	17.8	7.78	360.75	61.72	56.10	134.71	389.63	701.73	1.02	-	-
2014YJ-14	d	110º18´49"	34º47´51"	150	19.0	7.80	26.58	67.49	29.07	23.04	42.27	329.51	0.36	-	-
2014YJ-15	d	110º20´05"	34º48´02"	180	17.0	7.97	15.73	70.22	11.96	8.86	21.52	274.59	0.26	-	-
2014YJ-16	d	110º20´35"	34º50´31"	200	21.0	8.25	173.40	63.33	13.13	116.28	225.94	233.10	0.50	-	-
2014YJ-17	d	110º20´47"	34º50´02"	180	20.0	8.15	174.90	39.28	38.89	90.75	188.28	384.43	0.55	-	-
2014YJ-18	d	110º21´4"	34º52´37"	150	20.0	8.40	48.45	33.67	15.17	17.37	16.91	256.28	0.25	-	-
2014YJ-21	d	110º21´32"	34º54´38"	250	20.0	8.34	312.68	15.71	36.17	76.22	310.47	534.54	0.67	-	-
2014YJ-26	d	110º27´08"	34º54´39"	200	19.0	8.51	217.73	38.48	36.65	172.64	236.70	281.91	0.64	-	-
2014YJ-27	d	110º30´42"	34º54´01"	350	24.0	8.38	195.24	30.14	34.03	55.66	195.97	436.90	0.53	-	-
2014YJ-28	d	110º30´48"	34º55´34"	250	21.0	8.02	386.70	52.91	48.61	262.33	479.54	372.22	0.95	-11.34	-81.87
2014YJ-29	d	110º30´41"	34º55´52"	200	22.0	8.01	764.85	86.17	111.81	372.23	1431.33	396.63	1.51	-	-
2014YJ-30	d	110º27´32"	34º55´49"	300	19.0	8.35	513.65	32.06	57.12	409.09	545.64	351.48	1.20	-	-
2014YJ-31	d	110º37´47"	34º56´53"	280	22.0	7.95	582.53	86.17	88.47	232.55	1108.56	451.55	1.20	-9.97	-75.17

International Journal of
Environmental Research and Public Health

MDPI

Sample No.	Unit	Longitude	Latitude	Depth （m）	T （℃）	pH	Na (mg/L)	Ca （mg/L）	Mg （mg/L）	Cl (mg/L)	SO4 (mg/L)	HCO3 （mg/L）	TDS (g/L)	stable isotope δ18O (V-SMOW)‰	δD （V-SMOW） ‰
2014YJ-33	d	110º37′47"	34º58′03"	200	20.0	8.47	754.78	52.10	71.70	742.68	826.14	195.26	1.72	-	-
2014YJ-35	d	110º41′52"	34º58′13"	280	24.0	7.94	516.08	84.17	76.56	173.71	970.23	485.72	1.08	-9.67	-73.68
2014YJ-36	d	110º42′38"	34º58′40"	280	23.0	7.98	508.82	86.97	80.94	186.11	960.63	483.28	1.10	-	-
2014YJ-39	d	110º37′12"	34º51′02"	150	20.0	7.95	35.19	50.18	27.42	8.15	79.92	269.71	0.28	-	-
WX-01	d	111º8′17"	35º17′56"	280	20.0	8.26	330.55	32.06	32.64	303.54	206.5	227.32	1.15	-	-
WX-02	d	111º07′16"	35º17′57"	280	21.0	8.28	821.41	32.06	36.24	419.20	182.9	1373.02	2.31	-	-
WX-03	d	111º04′54"	35º20′37"	130	21.2	7.71	97.40	21.82	48.85	31.93	59.14	432.96	0.48	-	-
WX-07	d	111º18′3"	35º31′21"	210	18.9	8.19	196.47	16.63	25.32	17.56	63.95	596.63	0.63	-78.2	-10.57
WX-08	d	111º10′57"	35º26′50"	180	23.0	8.12	146.08	15.03	41.4	41.41	71.95	470.73	0.56	-	-
WX-09	d	111º17′39"	35º27′12"	130	17.2	8.24	109.55	20.04	22.32	17.87	27.72	394.49	0.40	-	-
WX-11	d	111º9′8"	35º27′3"	230	18.1	8.11	20.27	23.44	25.8	5.85	11.51	224.52	0.20	-	-
WX-12	d	111º23′24"	35º22′37"	140	18.8	7.91	21.07	32.06	24.48	11.97	17.51	232.22	0.23	-66.8	-8.93
WX-14	d	111º18′48"	35º19′26"	170	18.7	7.52	1019	355.9	511.6	1759	2370	292.37	6.19	-	-
WX-15	d	111º18′48"	35º19′26"	200	18.9	7.55	837.57	268.5	470.4	1402	2106	311.25	5.25	-	-
WX-16	d	111º9′49"	35º21′5"	245	20.0	8.10	237.10	31.86	42.36	132.6	354	257.40	0.95	-	-
XX-01	d	111º09′53.73"	35º10′36.11"	250	18.0	8.42	120.39	29.85	14.52	63.24	101.3	244.81	0.46	-78.6	-10.84
XX-03	d	111º09′0.69"	35º10′23.19"	260	19.0	8.10	153.26	28.25	35.4	50.45	182.1	343.43	0.63	-	-
XX-06	d	111º06′40.09"	35º14′22.49"	150	17.6	7.75	279.19	29.45	118.8	214.5	314	650.49	1.30	-	-
XX-07	d	111º05′51.23"	35º11′48.56"	260	17.2	8.26	65.90	18.03	29.76	38.83	72.72	210.53	0.34	-	-
XX-08	d	111º11′38.59"	35º14′51.00"	130	19.0	8.30	58.10	12.62	23.04	12.01	55.66	214.03	0.27	-	-
XX-09	d	111º11′57.50"	35º14′39.47"	240	19.1	8.16	133.01	22.04	28.08	36.48	76.15	398.69	0.50	-	-
XX-10	d	111º16′16.92"	35º16′38.96"	280	23.3	8.24	103.93	29.05	23.16	74	114.6	208.44	0.45	-74.8	-10.45
XX-11	d	111º18′59.62"	35º15′36.66"	210	19.2	7.94	124.92	42.08	13.44	11.65	22.03	476.32	0.46	-	-
XX-13	d	111º18′19.62"	35º14′5.55"	250	19.0	7.96	140.62	40.48	43.08	70.28	139.1	417.57	0.65	-	-
XX-15	d	111º16′30.32"	35º11′23.56"	150	18.5	7.98	110.11	43.68	30.12	41.35	90.61	387.49	0.51	-70.4	-9.53
XX-16	d	111º14′56.33"	35º10′30.44"	210	19.4	8.05	235.10	36.67	12.96	86.1	80.93	549.77	0.73	-	-
XX-17	d	111º14"3.46"	35º06′49.61"	150	39.0	8.16	1007	105.4	13.68	1191	492.6	440.65	3.07	-76.9	-10.41
XX-22	d	111º11"24.51"	35º04"15.01"	220	18.1	7.83	54.34	30.86	39.96	17.62	30.21	372.11	0.37	-	-
XX-24	d	111º07"31.12"	35º06"37.47"	300	20.9	8.48	443.92	32.06	23.04	352.6	253	456.04	1.34	-	-

International Journal of Environmental Research and Public Health

MDPI

Table S2. Pearson correlation coefficients of major hydrochemical parameter in shallow groundwater.

	EC	TDS	pH	K^+	Na^+	Ca^{2+}	Mg^{2+}	Cl^-	SO_4^{2-}	HCO_3^-	NO_3^-
EC	1.00										
TDS	0.86	1.00									
pH	0.06	0.08	1.00								
K^+	0.00	0.00	0.02	1.00							
Na^+	0.84	0.94	0.02	0.01	1.00						
Ca^{2+}	0.03	0.07	0.39	0.00	0.00	1					
Mg^{2+}	0.52	0.62	0.18	0.00	0.39	0.29	1				
Cl^-	0.87	0.86	0.11	0.00	0.74	0.17	0.68	1			
SO_4^{2-}	0.77	0.97	0.07	0.01	0.87	0.08	0.66	0.77	1		
HCO_3^-	0.42	0.41	0.01	0.00	0.56	0.04	0.07	0.26	0.30	1	
NO_3^-	0.12	0.07	0.00	0.01	0.07	0.01	0.06	0.06	0.05	0.13	1

Table S3. Pearson correlation coefficients of major hydrochemical parameter in intermediate-deep groundwater.

	EC	TDS	pH	K^+	Na^+	Ca^{2+}	Mg^{2+}	Cl^-	SO_4^{2-}	HCO_3^-	NO_3^-
EC	1.00										
TDS	0.82	1.00									
pH	0.02	0.05	1.00								
K^+	0.09	0.02	0.00	1.00							
Na^+	0.72	0.96	0.03	0.04	1.00						
Ca^{2+}	0.75	0.67	0.07	0.03	0.53	1					
Mg^{2+}	0.84	0.75	0.05	0.00	0.58	0.73	1				
Cl^-	0.75	0.53	0.01	0.13	0.42	0.64	0.68	1			
SO_4^{2-}	0.66	0.95	0.05	0.00	0.93	0.58	0.63	0.32	1		
HCO_3^-	0.34	0.05	0.00	0.00	0.02	0.13	0.04	0.05	0.08	1	
NO_3^-	0.03	0.03	0.02	0.00	0.08	0.00	0.00	0.02	0.00	0.14	1

Table S4. Variance explained by the first three principal components in shallow groundwater samples.

Component	Eigenvalue	% total variance	% cumulative
1	3.988	56.968	56.968
2	1.403	20.050	77.017
3	1.019	14.556	91.573

Table S5. Variance explained by the first three principal components in intermediate-deep groundwater samples.

Component	Eigenvalue	% total variance	% cumulative
1	4.220	60.280	60.280
2	1.088	15.546	75.826

© 2020 by the authors. Submitted for possible open access publication under the terms and conditions of the Creative Commons Attribution (CC BY) license (http://creativecommons.org/licenses/by/4.0/).
